# Fully ambulatory robotic single anastomosis duodeno-ileal bypass (SADI): 40 consecutive patients in a single tertiary bariatric center

**DOI:** 10.1186/s12893-024-02461-9

**Published:** 2024-07-09

**Authors:** Anne-Sophie Studer, Henri Atlas, Marc Belliveau, Amir Sleiman, Alexis Deffain, Pierre Y Garneau, Radu Pescarus, Ronald Denis

**Affiliations:** 1 Department of bariatric, robotic and minimally invasive surgery, Hôpital du Sacré-Coeur de Montréal, Université de Montréal, 5400 boul. Gouin ouest Montréal, Québec, H4J 1C5 Canada; 2 Department of anesthesiology, Hôpital du Sacré-Coeur de Montréal, Université de Montréal, 5400 boul. Gouin ouest Montréal, Québec, H4J 1C5 Canada

**Keywords:** Ambulatory surgery, Same day discharge (SDD), Obesity epidemic, Single anastomosis duodeno-ileal bypass (SADI), Robotic bariatric surgery

## Abstract

**Background:**

Single Anastomosis Duodeno-Ileal bypass (SADI) is becoming a key option as a revision procedure after laparoscopic sleeve gastrectomy (LSG). However, its safety as an ambulatory procedure (length of stay < 12 h) has not been widely described.

**Methods:**

A prospective bariatric study of 40 patients undergoing SADI robotic surgery after LSG with same day discharge (SDD), was undertaken in April 2021. Strict inclusion and exclusion criteria were applied and the enhanced recovery after bariatric surgery protocol was followed. Anesthesia and robotic procedures were standardized. Early follow-up (30 days) analyzed postoperative (PO) outcomes.

**Results:**

Forty patients (37 F/3 M, mean age: 40.3yo), with a mean pre-operative BMI = 40.5 kg/m^2^ were operated. Median time after LSG was 54 months (21–146). Preoperative comorbidities included: hypertension (*n* = 3), obstructive sleep apnea (*n* = 2) and type 2 diabetes (*n* = 1). Mean total operative time was 128 min (100–180) (mean robotic time: 66 min (42–85)), including patient setup. All patients were discharged home at least 6 h after surgery. There were four minor complications (10%) and two major complications (5%) in the first 30 days postoperative (one intrabdominal abscess PO day-20 (radiological drainage and antibiotic therapy) and one peritonitis due to duodenal leak PO day-1 (treated surgically)). There were six emergency department visits (15%), readmission rate was 5% (*n* = 2) and reintervention rate was 2.5% (*n* = 1) There was no mortality and no unplanned overnight hospitalization.

**Conclusions:**

Robotic SADI can be safe for SDD, with appropriate patient selection, in a high-volume center.

## Introduction

Current findings support the role of metabolic and bariatric surgery in lowering the costs associated with common comorbidities such as diabetes mellitus, hypertension, dyslipidemia, and heart disease [1]. Obesity is increasingly prevalent worldwide [2–4] and the advent of the COVID19 pandemic [5, 6] postponed elective bariatric surgeries, leading to increased wait times for patients [7–9]. Same day discharge (SDD) bariatric surgery may be part of the answer with simpler procedures and fewer care requirements [10–15].

Since the inception of our bariatric surgery SDD program in 2012, the results of ambulatory Laparoscopic Sleeve Gastrectomy (LSG) procedures were very positive [16]. More than half of the 600 LSG performed on average in our institution every year are now SDD. This is possible because of stringent preoperative patient selection and an easy access to postoperative care when complications arise. Since 2016 over 300 Bilio-Pancreatic Diversion (BPD) surgeries were done in our center [17], including Single Anastomosis Duodeno-Ileal bypasses (SADI). Most were performed for weight regain (60%) or insufficient weight loss (25%), or because initially planned as a two-stage procedure after LSG (15%); 40% of those procedures were robot-assisted surgeries. Based on this experience, we offered SADI after LSG with SDD to patients meeting the same preoperative selection criteria and with access to the same postoperative care in case of complications [16]. This strategy reduced the wait time for this category of patients.

A recent systematic review reported a 5.7% revision rate 2 years after LSG and 75.6% after 6 years [18]. Meanwhile, SADI is becoming a key option for revision following LSG [19]. SADI is a simplified version of the Duodenal-Switch (BPD-DS) [20–22]. Having only one anastomosis reduces operative time and lowers the complication rate compared to BPD-DS [23], while offering more weight loss than Roux-en-Y gastric bypass (RYGB) [23, 24]. Its safety as a SDD procedure (length of stay < 12 h) has yet to be proven. We aimed to assess the early outcomes (30-day morbidity-mortality, emergency department visits, readmission rate, reintervention rate) of patients undergoing robot-assisted SADI after LSG with SDD.

## Materials and methods

### Study design

This is a retrospective analysis of a prospectively collected database covering the period between April 2021 and December 2022, and including 40 patients who underwent robot-assisted SADI after LSG (length of stay < 12 h). There are no hospitalization facilities at our center, however, the three surgeons are affiliated with a nearby tertiary bariatric center hospital with available transportation and inpatient admission. Surgeries were scheduled first in the morning, and no more than two cases could be performed daily, to allow patient discharge before 7:00 p.m.

### Patient selection

Patients had to meet strict selection criteria to be eligible (Table 1) [25, 26]. Preoperative assessment included full blood count, renal function and electrolytes, thyroid and parathyroid function, hepatic enzymes, albumin, and proteins, lipid panel, coagulation status (INR and PTT), glycemia and glycated hemoglobin, iron level, and vitamins (folic acid, vitamin D, and B12). All patients included for SDD had normal hemoglobin and no liver or renal disease, and any vitamin deficiency was treated before surgery. All patients had a preoperative upper gastrointestinal endoscopy. Patients had to follow a pre-operative low-calorie diet (2 to 4 weeks depending on initial BMI), ensuring an intake of 900 kcal and 90 g of protein per day, to reduce liver size and facilitate intra-operative exposure. All patients took a preoperative nutrition class and received counseling.

**Table 1:** Eligibility criteria for ambulatory management

Inclusion criteria	Exclusion criteria
Age < 55 yo with BMI ≤ 50 kg/m^2^ Age < 45 yo with BMI ≥ 50 and < 55 kg/m^2^ ASA score I or II, or III if cleared by internist, Moderate or severe obstructive sleep apnea syndrome if well controlled with CPAP Obesity Surgery Mortality Risk [24, 25] score grade A or B Residence within 40 km from hospital	Age ≥ 55yo and BMI > 50 kg/m^2^ Age ≥ 45 yo and BMI ≥ 55 kg/m^2^ ASA score ≥ IV Obesity Surgery Mortality Risk [24, 25] score grade C Insulin-dependent diabetes Poorly controlled hypertension Complex previous abdominal surgeries

### Anesthesia protocol

Enhanced recovery after bariatric surgery (ERABS) protocols play an essential role in patients’ outcomes and clearly demonstrate the importance of having an experienced anesthetic team following guidelines [27]. We currently encourage all patients to drink carbohydrate rich liquids without pulp up to 2 h prior to surgery. Preoperative analgesia was initiated with 1000 mg of acetaminophen and 400 mg of celecoxib, unless contra-indicated. Although still controversial, most anesthesiologists involved follow an opioid free/opioid sparing protocol (Table 2.), favoring synergistic nociceptive pathways and short action anesthetics [28]. Dexamethasone (10 mg) is used preventively to reduce nausea, vomiting, pain and opioid consumption [29–31]. Similarly, 4 mg of ondansetron is always given postoperative to prevent nausea and control vomiting. Deep muscle relaxation offers better surgical outcomes [32], and since the cost-benefit issue is no longer a concern sugammadex was administered for proper reversal and diminished morbidity [33, 34]. Postoperative analgesic was reinforced by the subcutaneous injection of 20 ml of bupivacaine at 0.5% of the port scar incisions [35, 36]. All patients were given 1 L of crystalloids at the beginning of surgery followed by another liter administered gradually during the surgical procedure and in the recovery room.

**Table 2:** Institution’s protocol for ambulatory intraoperative medication and management

Anesthesia Protocol	Surgical Protocol	Recovery Room Protocol	Discharge Protocol
**Induction**: Propofol 200-400 mg Ketamine 0.5 mg/kg Dexmedetomidine 0.3–0.5 mcg/kg Lidocaine 2 mg/kg Magnesium 30 mg/kg **Maintenance**: Sevoflurane(1MAC) or BIS guided TIVA* **Muscle relaxant**: Rocuronium (70-120 mg) **Reverse**: Neostigmine 2.5 mg-4 mg) Glycopirolate 0.5 mg-1.2 Or Sugammadex 2 mg/kg **Narcotics**: Dilaudid 0.5–1.5 Morphine 2-5 mg Fentanyl 0-150mcg **Antiemetics**: Ondansetron 4 mg Dexamethasone 10 mg **Cristalloids**: Bolus 15 cc/kg	**Antibiotic prophylaxis**: Cefazolin 2 g **Antithrombotics**: Heparin 5000 UI SC before surgery **Intermittent compression stockings** **2 experimented surgeons available** **Standardised** Laparoscopic & Robotic technique **Local anesthesia**: Bupivacaïne 0.5%	**Vital Signs** **Intermittent compression stockings** **PPI**: Pantoloc 40 mg **Analgesia**: Acetaminophen 975 mg Hydromorphone 1-2 mg **Antiemetics**: Dimenhydrate 50 mg Ondansetron 4 mg	PACU** modified criteria: score > 10/14 **Prescription**: Enoxaparine 40 mg daily Hydromorphone 1 mg every 6 h if needed (max 7days) Dimenhydrinate 50 mg (every 6 h if needed max 7 days) Docusate sodium 200 mg (twice a day, if needed max 7 days) Pantoprazole 40 mg daily, for 1 month) **Vitamin supplements** **Telephone contact 24 h post-op**

* Bispectral Index Monitoring guided Total Intravenous Anesthesia ** Post−Anesthesia Care Units

### Surgical procedure

The patient was in the supine position, legs spread and under general anesthesia. A 6-trocarts (including 2 robotic ports) standardized laparoscopic technique was initiated. After assessing the sleeve gastrectomy, we measured 250 cm of bowel from the ileo-caecal valve laparoscopically and confirmed it could easily be sutured to the duodenum without tension. Then, the robot was used for the following steps and retroduodenal dissection completed using the vessel sealer. The duodenum was transected 2 to 3 cm distal to the pylorus, using a stapler device and reinforced with a suture over the duodenal stump. A sero-muscular suture between the duodenum and the ileum at 250 cm, as previously measured, was made to relieve any tension. Then, a termino-lateral duodeno-ileal anastomosis was performed in a single running posterior layer, followed by an anterior layer, using 3 − 0 absorbable V-lock. The anastomosis was 2 to 2.5 cm wide. It is also the norm at our institution to perform a methylene blue test via a nasogastric tube to rule out any mechanical leak at the end of the procedure; when needed an abdominal drain was left in place close to the anastomosis. The presence of gastric reflux managed with proton pump inhibitor (PPI) or of a hiatal hernia found during the preoperative endoscopic study were not an absolute contraindication to SADI. However, any existing hiatal hernia was repaired, if necessary, by a primary crural repair (using non resorbable suture for closing the crus anteriorly and posteriorly).

### Postoperative course

Patients were released home in accordance with the modified Post-Anesthesia Care Units (PACU) discharge protocol [37]. These patients were eupneic, mobile, and well-oriented, with normal blood pressure and oxygen saturation, normal urine, and clean dressing, with pain and nausea controlled through oral medication. Neither postoperative imaging nor blood tests were planned. Oral medication at discharge included thromboprophylaxis with low molecular weight heparin for 21 days (standard protocol at our institution considering that our patients suffer from obesity, are placed in the Fowler position during a prolonged period of time, and go through a major surgical stress), analgesic, antiemetics, laxatives and PPI for 4 weeks postoperative and vitamins (Table 2). Refeeding guidelines were strict and liquid intake fractionated. Follow-up included a telephone call PO day-1 and visits at the clinic within the first week if an abdominal drain needed to be removed (timing for removal was decided by the surgeon who treated the patient) and at 1, 6 and 12 months thereafter (Table 2). Demographic and medical characteristics of patients, intraoperative details (total operative time i.e., induction of anesthesia, positioning the patient, incision, laparoscopic step (common channel count), docking the robot, robot-assisted surgery, removing the robot and skin closure), 30-day morbidity-mortality according to Dindo-Clavien’s classification [38], emergency department visits, readmission rate and reintervention rate were also analyzed. The results are presented as means and standard deviations or counts (%) as appropriate, and medians (min-max). According to local requirements and guidelines, with approval from hospital management, this retrospective review of a prospectively collected database did not require informed patient consent.

## Results

Ambulatory robot-assisted SADI was performed on 40 patients including three males and 37 females, with a mean age of 40.3 (± 7.7) years. All patients had previously undergone LSG as SDD (67.5% *n* = 27) or within 24 h of hospitalisation (32.5% *n* = 13). Mean BMI for these patients was 47.7(± 7.1) kg/m^2^ before LSG and 40.5(± 4.8) kg/m^2^ before SADI. All patient characteristics and comorbidities are described in Table 3. SADI was indicated for either weight regain or insufficient weight loss after LSG, in respectively 65% (*n* = 26) and 35% (*n* = 14) of cases. Intra-abdominal drainage was used after the procedure because of difficult retroduodenal dissection in 10 cases and because of an inconclusive leak test in four others (difficult placement of the nasogastric tube in the antrum for the test). There was no unplanned overnight stay.

**Table 3:** Demographic characteristics of the study population

Characteristics	*n* counts (%) = 40 patients
**Gender** M/F **Mean Age** (yo) **Pre-operative BMI** (kg/m^2^)	3/37 (7.5/92.5%) 40.3 (±7.7) min-max (28-58) 40.5 (±4.8) min-max (31.6-49.1)
**Pre-operative comorbidities before SADI**	
Hypertension Obstructive Sleep Apnea Syndrome Dyslipidemia Type2 Diabetes Recurrence of comorbidities after LSG Persistence of comorbidities after LSG	3 (7.5%) 2 (5%) (using CPAP) 0 (0%) 1 (2.5%) (Semaglutide, preop HbA1c=5.4%) 4(10%) 2(5%)
**Bariatric history**:	
Gastric lapband Gastric plication LSG Ambulatory /Overnight hospitalisation Median delay between SG and SADI Mean pre-operative BMI before SG	7 (17.5%) 1 (2.5%) 40 (100%) 27/13 (67.5/32.5%) 54 months (min-max: 21-146) 47.7 (±7.1) min-max 31-66
**Indication for SADIs**:	
Insufficient weight loss Weight regain	14 (35%) 26 (65%)
**Per-operative details**	
Robotic SADI Concomitant hiatal hernia repair Abdominal drainage: Inconclusive leak test Difficult retroduodenal dissection	40 (100%) 8 (20%) 14 (35%) 4 10
Mean operative time (min):	
Total Robotic part Robotic docking	128 (min-max: 100-180) 66 (min-max:42-85) 6min45 (min-max: 4min8-10)
Mean stay in recovery room VAS ≤4/10 at time of discharge	5h45 (min-max: 4h35-6h55) 40 (100%)

BMI: Body Mass Index/ CPAP: continuous positive airway pressure/ LSG: Laparoscopic Sleeve Gastrectomy / SADI: Single Anastomosis Duodeno−Ileal bypass/ VAS: Visual Analog Scale

**Fig. 1:**
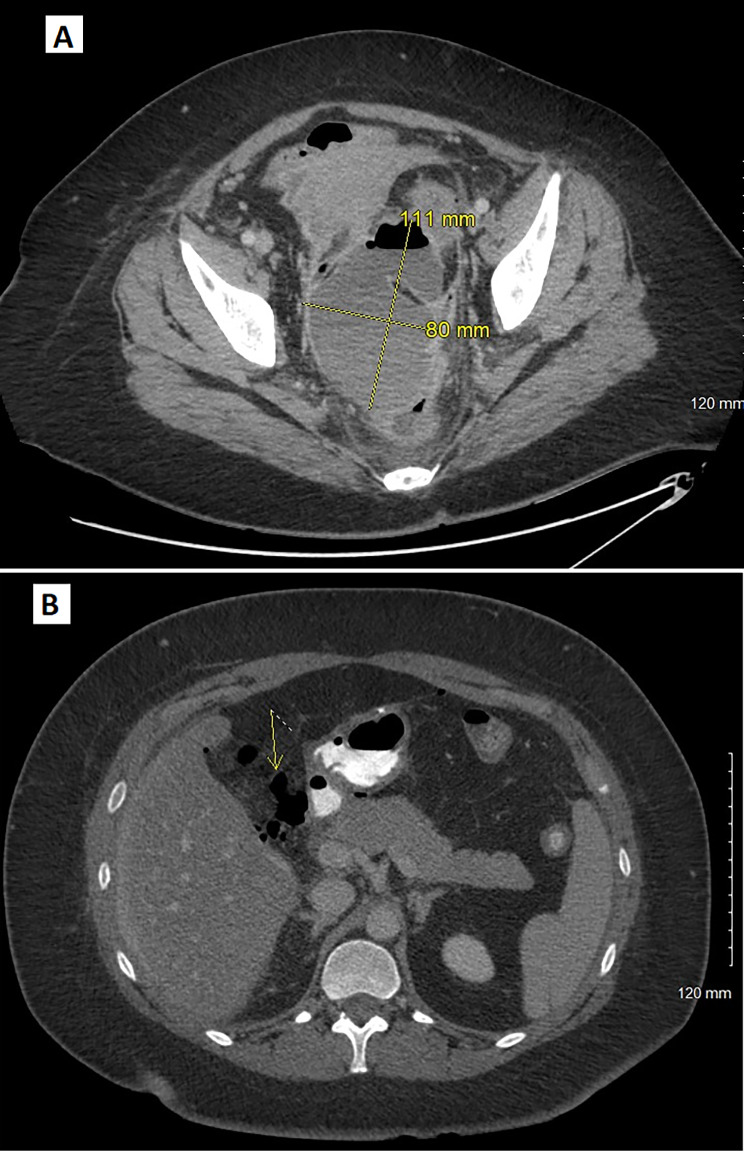
Major post-operative complications. **a**: 45yo patient, PO day-20, CT scan showed a pelvic collection compatible with infected hematoma (Dindo-Clavien IIIa). **b**: 34yo patient, PO day-1, CT scan showed a proximal duodenal leak (Dindo-Clavien IIIb)

Two patients (5%) were readmitted because of major complications (Tables 4 and 5). The first was a 45-year-old female, readmitted 20 days PO, for abdominal pain. Retroduodenal dissection was difficult due to severe pancreaticoduodenal adhesions. A drain was left in place and removed on day 7. The CT scan showed a pelvic collection compatible with infected hematoma (Fig. 1A). Treatment consisted of antibiotic therapy and percutaneous radiological drainage (Dindo-Clavien IIIa). The second case involved a 34-year-old patient, readmitted the day after surgery for abdominal pain. The readmission abdominal-pelvis CT scan showed a proximal duodenal leak (Fig. 1B). However, the drain still in place did not release any turbid fluid. Surgical findings revealed a tear on the anastomosed duodenum, probably secondary to excessive intraoperative traction. A suture with Graham patch was performed (Dindo-Clavien IIIb). Four patients experienced minor complications (10%) that were treated at the emergency department. According to these patients, emergency consultation occurred early after surgery (median: 4.5 ± 3.5, min-max:1–8 days) because of abdominal pain. Lab tests and abdominopelvic CT scan with oral contrast were performed for each patient to confirm there were no underlying complications. One of these patients with an abdominal drain had parietal cellulitis along the drain path and was treated with antibiotics and drain removal (Dindo-Clavien II), on day 7 PO. All four patients were given pain relief and support and did not require hospitalization. There was no mortality.

**Table 4:** Morbidity, mortality, and readmission rates

Morbidity Rate	*n* counts (%)
Emergency department visits: Minor complications: Major complications: *Readmission rate*: *Reintervention rate*:	6 (15%) 4 (10%) 2 (5%) 2 (5%) 1 (2.5%)
**Mortality rate**	0 (0%)

**Table 5:** Dindo-Clavien’s classification of surgical complications

Grade	Type of complication	Length of stay (days)	*n* counts (%)
I	Abdominal pain	0	3 (7.5%)
	Nausea and vomiting	*NA*	0 (0%)
II	Parietal cellulitis	0	1 (2.5%)
IIIa	Infected intra-abdominal hematoma	10	1 (2.5%)
IIIb	Duodenal leak and peritonitis	24	1 (2.5%)

NA: Not Applicable

## Discussion

There is a growing interest in ambulatory care, made more pressing since the coronavirus (COVID 19) pandemic. Indeed, several patients wish to minimize their hospital stay while having quick and safe access to surgical care.

A recent ACS-NSQIP analysis of 36 042 patients showed an early readmission rate after bariatric surgery of 4.7%, (gastric adjustable lap band, sleeve gastrectomy and gastric bypass procedures in a non ambulatory settings) mostly due to upper GI symptoms (12.95%) [39]. Another study on 437 patients with primary SADI surgery, outside the ambulatory setting, reported a morbidity rate of 7.7% at 30 days, with readmission and reoperation rates of 1.8% and 1.3%, respectively [40]. The most common complication was nausea (*n* = 10, 2.2%). Meanwhile, a study on 328 ambulatory LSG, reported that 4.9% of patients were readmitted for nausea/vomiting postoperatively [16]. Another study on 82 ambulatory SADI-S patients noted nausea and dehydration in five patients (6.1%), although they all received planned intravenous fluid therapy within 3 days after the primary procedure [41]. This was not the case in our ambulatory SADI patients. The studies cited all had full stapling of the stomach, which was not done in our patients. Hence, these side-effects should not be considered contraindications to SDD. Furthermore, as recommended in the ERABS protocol patients could drink liquids until 2 h preoperatively. They also received weight-dose intravenous crystalloid fluid therapy during and after surgery. Finally, perioperative drugs were chosen to prevent and limit the occurrence of nausea and vomiting and to promote a rapid resumption of oral feeding.

Some of the main concerns in ambulatory bariatric surgery are leaks and bleeding. Nonetheless, relatively low complication rates have been reported for RYGB patients with SDD; 0.3% and 0.2% gastrointestinal bleeding and gastro-jejunal leaks, respectively, in one study [42] and up to 4% in other studies, mostly leaks [40–44]. We recently published data on major morbidity after 2nd stage SADI (without SDD) and the rate of duodenal-ileal anastomotic leaks was 2.7% [17]. Conversely, Garofalo et al. and Al-Masrouri et al. had low postoperative bleeding rates, 0.3% and 1.7% respectively, with ambulatory LSG patients [15, 16]. In the present study the readmission rate was explained by technical considerations. One case of difficult retroduodenal dissection with a postoperative hematoma that got infected and another case of serotomy with an early leak. Both had intrabdominal drains at the time of SADI which did not reveal or drain the infected liquid. Another patient developed cellulitis along the drain and another case of PO abdominal pain was due to the presence of the drain. Being among the first to implement SDD SADI meant taking extra precautions to avoid early complications (such as bleeding or leaks), even if drainage was not necessarily part of the ERABS protocol. Nonetheless, it is now avoided as often as possible. Sanchez-Pernaute et al. published their preliminary findings on 16 revision SADI patients after LSG, with zero early morbidity [21]. Given the data from high-volume centers, ambulatory protocols for this type of surgery may be indicated.

The limitation of our study is in its unicentric, retrospective, and non-randomised design. These are the early results on a small sample of young patients with few comorbidities undergoing revision SADI surgery. The early outcomes are satisfactory, suggesting that this is an avenue worth exploring but with a larger dataset that will also include laparoscopic cases. We deliberately chose to include only robotic surgeries for homogeneity and because it is our preferred technique for revisional surgery. The standard limitations with laparoscopy may be heightened by the complex anatomy of patients suffering from obesity and the challenges inherent to revision procedures. These can be minimized with robot-assisted surgery which offers ergonomic, three-dimensional high-definition view, direct camera control by the surgeon, multiquadrant access, tremor filtration, and endowristed instruments which improve the accuracy of some complex laparoscopic tasks like suturing; this may influence morbidity rates [45]. Our complication rate is acceptable, suggesting that outpatient management may be possible for centers with the appropriate facilities.

Studies have shown that well-designed ambulatory programs must follow the three ERABS protocols (pre, intra and postoperative) with a focus on multidisciplinary/parallel teamwork at all levels, as well as intensive counseling and education throughout the patient’s journey [15, 41, 46]. It should be noted that several conditions have to be met to achieve SDD: surgery performed by an experienced team, a high-volume bariatric center, strict selection criteria, a multimodal approach, and a postoperative emergency safety plan. This can be achieved in high-volume centers where management is the same for all patients and with the entire team following the same guidelines. Centers with a high volume of bariatric surgery and SDD procedures that follow a codified strategy can anticipate complications, thus contributing to a lower readmission rate [47, 48].

## Conclusion

This study found acceptable early outcomes for patients undergoing ambulatory robotic SADI (length of stay < 12 h), when performed by an experienced team with appropriate eligibility criteria. In order to improve patient access to metabolic and bariatric care, the outpatient setting could be used not only for primary LSG but also for revisional SADI.

## Data Availability

The datasets used and/or analysed during the current study are available from the corresponding author on reasonable request.
